# Advantages of skeletonizing the left colic artery in low anterior resection cases related to vascularity with ICG: A systematic review

**DOI:** 10.1097/MD.0000000000044491

**Published:** 2026-01-30

**Authors:** Sarim Rashid, Muhammad Zeeshan, Almaz Rehman, Muhammad Adeel, Abida Parveen

**Affiliations:** a Department of Medicine, Ibn e Seena Hospital, Kabul, Afghanistan.

**Keywords:** anastomotic leak, bowel perfusion, colorectal cancer, fluorescence imaging, indocyanine green, left colic artery, Low anterior resection

## Abstract

**Background:**

LAR for rectal cancer requires optimal bowel perfusion to minimize AL, a serious postoperative complication. Preservation of the left colic artery (LCA) through skeletonization may improve vascular supply. Indocyanine green (ICG) fluorescence imaging is increasingly utilized intraoperatively to assess real-time bowel perfusion and guide surgical decisions.

**Methods:**

A systematic review was conducted following PRISMA guidelines. Databases including PubMed, Scopus, Web of Science, and Embase were searched up to May 2025. Studies evaluating intraoperative ICG fluorescence imaging in colorectal cancer surgery with outcomes related to AL, lymph node yield, or recurrence were included. Both randomized and observational studies were considered. Quality was assessed using the Newcastle-Ottawa Scale, and a qualitative synthesis was performed.

**Results:**

Nine studies involving 1771 patients met inclusion criteria. ICG fluorescence imaging consistently improved intraoperative assessment of bowel perfusion, influencing surgical decision-making and reducing AL rates, particularly when combined with LCA skeletonization. Preservation of the LCA was associated with enhanced vascular integrity and comparable lymph node yields. Studies reported an AL reduction from approximately 10% to below 5% with ICG guidance. ICG also aided in optimizing transection lines and improved lymphadenectomy precision.

**Conclusion:**

ICG fluorescence imaging, combined with LCA skeletonization, appears to enhance bowel perfusion assessment and reduce anastomotic complications in LAR for colorectal cancer. While promising, further randomized controlled trials are needed to confirm long-term oncologic outcomes and establish standardized protocols.

## 1. Introduction

Low anterior resection (LAR) is a widely performed surgical procedure for rectal cancer, aiming to achieve oncologic clearance while preserving bowel continuity and function. A critical determinant of postoperative outcomes, particularly anastomotic healing, is adequate perfusion of the proximal and distal bowel segments.^[1]^ Insufficient vascular supply remains one of the most significant risk factors for anastomotic leakage (AL), a complication associated with increased morbidity, mortality, and impaired oncologic outcomes. Therefore, techniques that optimize colonic perfusion during LAR are of increasing clinical interest.^[2]^

The left colic artery (LCA), arising from the inferior mesenteric artery (IMA), is a major source of blood supply to the descending and sigmoid colon. Traditional high ligation of the IMA sacrifices the LCA in favor of a more extensive lymphadenectomy, but this may compromise blood flow to the anastomotic site.^[3]^ In contrast, skeletonization of the LCA allows preservation of the artery while still achieving adequate oncologic resection. This technique may enhance vascular integrity of the proximal colon, thereby potentially reducing anastomotic complications.^[4]^

Indocyanine green (ICG) fluorescence angiography is increasingly used intraoperatively to assess tissue perfusion in real time. The application of ICG imaging enables objective evaluation of the vascular benefit provided by LCA skeletonization, guiding intraoperative decisions and improving patient safety.^[5]^ Despite growing adoption, the advantages of LCA skeletonization in the context of vascularity assessment using ICG remain to be systematically evaluated.^[6]^

The rationale of the review is to synthesize and critically evaluate the growing body of evidence on the combined use of LCA skeletonization and ICG fluorescence imaging during LAR for colorectal cancer. While both techniques are increasingly adopted to enhance intraoperative decision-making and reduce anastomotic complications, their synergistic impact on bowel perfusion, AL, lymph node yield, and oncologic safety remains incompletely defined. This review aims to clarify their combined benefits, assess the consistency of outcomes across studies, and identify gaps in evidence to inform future clinical practice and research.

## 2. Methods

### 2.1. Protocol and registration

**Figure 1.**
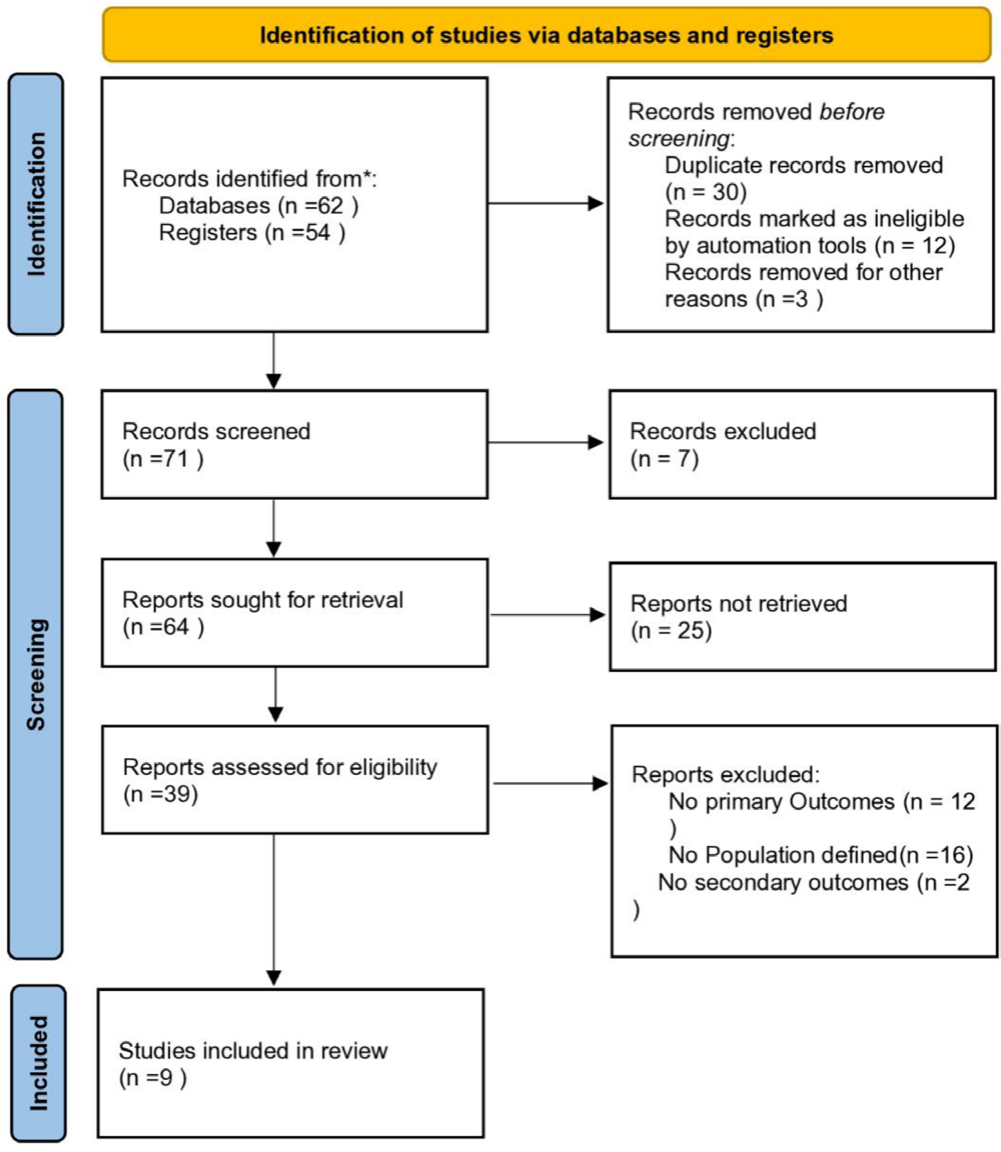
Preferred Reporting Items for Systematic Reviews and Meta-Analyses flowchart.

This systematic review was conducted in accordance with the Preferred Reporting Items for Systematic Reviews and Meta-Analyses (PRISMA) guidelines (Fig. 1). A predefined protocol outlining the objectives, inclusion criteria, and methodology was developed prior to the literature search.

### 2.2. Eligibility criteria

Studies were included if they involved adult patients undergoing colorectal cancer surgery with intraoperative use of indocyanine green (ICG) fluorescence imaging for vascular or perfusion assessment. Eligible study designs included randomized controlled trials, prospective cohort studies, retrospective cohort studies, and case-control studies. Studies were required to report at least one of the following outcomes: AL rate, recurrence rate, or lymph node yield. Exclusion criteria were case reports, reviews, editorials, letters, conference abstracts, animal studies, and non-English language publications.

### 2.3. Search strategy

A comprehensive literature search was conducted in PubMed, Scopus, Web of Science, and Embase databases upto may 2025. The search strategy included combinations of keywords and Medical Subject Headings (MeSH) such as “colorectal cancer,” “indocyanine green,” “ICG,” “fluorescence imaging,” “anastomotic leak,” and “perfusion assessment.” Boolean operators (AND/OR) were applied to enhance search sensitivity.

### 2.4. Study selection

Two independent reviewers screened the titles and abstracts for relevance. Full texts of potentially eligible studies were then reviewed to determine final inclusion. Discrepancies between reviewers were resolved through discussion or consultation with a third reviewer. “skeletonization of the LCA” refers to the meticulous dissection and preservation of the LCA while removing surrounding lymphatic and connective tissue, including perivascular nodes, without compromising arterial integrity. This definition is adopted to standardize interpretation across studies where the term is variably used. It allows for adequate oncologic clearance while maintaining perfusion to the distal bowel, thereby potentially reducing AL.

### 2.5. Data extraction

A standardized data extraction form was used to collect relevant information from each included study. Extracted data included: author, publication year, study design, sample size, patient demographics, type of colorectal cancer, type of intraoperative ICG assessment, anastomotic leak rate, recurrence rate, lymph node yield, diagnostic imaging methods, and reported clinical or oncologic outcomes. Data extraction was conducted independently by 2 reviewers to ensure accuracy.

### 2.6. Quality assessment

The quality of included non-randomized studies was assessed using the Newcastle-Ottawa Scale, which evaluates studies based on 3 domains: selection of study groups, comparability, and ascertainment of outcomes. Each study was independently scored by 2 reviewers, and discrepancies were resolved by consensus. Studies scoring 7 or more points were considered high quality.

### 2.7. Data synthesis

Due to heterogeneity in study designs, outcome measures, and lack of consistent comparative data, a qualitative synthesis was performed rather than a meta-analysis. Findings were grouped and analyzed according to study characteristics and reported outcomes, with a focus on the impact of ICG fluorescence imaging on intraoperative decision-making, anastomotic leak rates, lymph node yield, and overall surgical outcomes.

## 3. Result

A total of 9 studies involving 1771 participants were included in this systematic review, evaluating the intraoperative application of indocyanine green (ICG) fluorescence imaging in colorectal cancer surgery. Across these studies, ICG was consistently shown to enhance surgical decision-making and clinical outcomes.

Wang et al (2023) retrospectively analyzed 183 patients with proctosigmoid colon cancer and demonstrated that ICG-guided IMA angiography effectively identified poorly perfused anastomotic regions, allowing for better vascular assessment and reduced ischemic risk.^[7]^ Yanagita et al (2021), in a study with 384 patients (197 with ICG and 187 controls), found a significantly lower anastomotic leak (AL) rate in the ICG group (3.3%) compared to the control group (10.7%), emphasizing the role of ICG in intraoperative perfusion evaluation.^[8]^

Liu et al (2023) assessed 194 patients and reported that low ligation of the IMA, preserving the LCA, resulted in slightly lower AL rates (21% vs 24%) and comparable lymph node yields, supporting the benefit of ICG-guided vascular strategy in sigmoid and rectal cancer.^[9]^ Similarly, Qiu et al (2025) in a prospective study of 193 patients, demonstrated that fluorescence lymph node dissection significantly improved lymph node harvest and surgical precision in mid-low-rectal cancer.^[10]^

In an observational study of 110 patients, Chang et al (2019) found that ICG angiography led to changes in the surgical plan in many cases, particularly those with rectal cancer and marginal perfusion.^[11]^ Kawada et al (2017), with 68 participants, showed that ICG fluorescence imaging helped redefine the optimal transection line based on real-time perfusion visualization, reducing the risk of AL.^[12]^ Son et al (2019) confirmed this through quantitative analysis of perfusion using T1/2MAX and TR in 86 patients, aiding in identifying poorly perfused segments and minimizing complications.^[13]^

Boni et al (2016), in a cohort of 107 patients, reported that ICG fluorescence provided critical intraoperative information that influenced resection decisions, with only 1 patient developing AL.^[14]^ Lastly, Kim et al (2016) studied 436 patients (123 ICG vs 313 non-ICG), showing a lower AL rate in the ICG group, particularly in rectal cancer patients undergoing robotic-assisted sphincter-saving operations (RA SSO), where ICG enhanced real-time perfusion monitoring.^[15]^

Overall, the collective evidence supports that ICG fluorescence imaging improves intraoperative vascular assessment, reduces anastomotic leak rates, enhances lymph node yield, and contributes to better oncologic and clinical outcomes in colorectal cancer surgery. The characteristics and outcomes of the included studies are detailed in Table 1, which highlights variations in study design, sample size, cancer type, and clinical endpoints such as anastomotic leak and recurrence rates.

**Table 1:** Study characteristics.

Author, references	Year	Study design	Sample size	Intraoperative vascular assessment with ICG	Anastomotic leak rate	Recurrence rate	Type of cancer	Diagnostic imaging	Oncologic outcomes	Clinical outcomes
Wang et al^[7]^	2023	Retrospective cohort study	N = 183	–	–	–	PCC	IMA angiography	5.21% (5/96) patients had poor anastomosis at the primary arch, and secondary vascular arches (6.4 (4.6–10.0) cm from the intestinal wall) were observed in 38.54% of patients.	ICG guidance ensures optimal intestinal perfusion and reduces ischemic risk in laparoscopic PCC surgery.
Yanagita et al^[8]^	2021	Retrospective case-control study	N = 384(ICG group = 197, control = 187)	179 patients with good perfusion and 18 (9.1%) with poor perfusion	6 (3.3%) in ICG group, 17 (10.7%) in control	–	–	–	–	Intraoperative ICG fluorescence imaging may significantly reduce AL incidence.
Liu et al.^[9]^	2023	Retrospective case-control study	N = 194 (high ligation = 46 patients, low ligation = 148 patients)	–	Lower in low ligation group	In high ligation = 11(24%), in low ligation n = 32(21%)	Sigmoid or rectal cancer	–	The H-L group had an average of 17.4 lymph nodes detected per person (43% positive), while the L-L group had 15.9 (41% positive).	Preserving the LCA during mesenteric resection with lymph node dissection at the IMA root benefits laparoscopic colorectal cancer surgery.
Qiu et al^[10]^	2025	Prospective non-randomized controlled study	N = 193 (n = 129) with laparoscopic surgery, n = 64, with FLND	–	–	–	Mid-low-rectal cancer	–	FLND significantly increased lymph node yield.	FLND-assisted radical surgery improves lymphadenectomy accuracy and outcomes in mid-low-rectal cancer.
Chang et al^[11]^	2019	Observational study	N = 110 (sigmoid colon [29.1%] and rectum) (70.9%)		Overall anastomotic leakage rate = 5.5%.		Left-sided colorectal cancer	ICG fluorescence angiogram	51.8% of the cases involved total mesorectal excision	ICG fluorescence angiography often altered operative plans, especially in rectal cancer and poorly perfused colon cases.
Kawada et al^[12]^	2017	Prospective study	N = 68		Three patients (4.5 %)		Left-sided colorectal cancers	ICG fluorescence	Intestinal perfusion was present at 3 mm (median) distal to the initially planned transection line.	ICG fluorescence imaging aids transection line selection in laparoscopic colorectal DST anastomosis.
Son et al^[13]^	2019	Prospective study	N = 86		Anastomotic leak, n = 3		Colorectal cancer	Laparoscopic fluorescence imaging		Quantitative ICG analysis using T1/2MAX and TR helps identify poorly perfused segments, reducing anastomotic complications in laparoscopic colorectal surgery.
Boni et al^[14]^	2016	Prospective study	N = 107		One patient had an anastomotic leakage		Colorectal cancer	ICG-enhanced fluorescence		ICG angiography guides vascular perfusion assessment, potentially altering resection or anastomosis sites and reducing leak rates in colorectal surgery.
Kim et al^[15]^	2016	Prospective cohort study	N = 436 (n = 123 with ICG and n = 313 without ICG)		Was greater in without ICG group		Rectal cancer			ICG imaging during RA SSO offers real-time perfusion assessment, reducing AL risk in patients prone to bowel ischemia.

AL = anastomotic leak, ICG = indocyanine green, IMA = inferior mesenteric artery, PCC = proctosigmoid colon cancer, RA SSO = undergoing robotic-assisted sphincter-saving operations.

The included studies primarily focused on intraoperative and short-term clinical outcomes, such as improved perfusion assessment, reduced anastomotic leak rates, and enhanced lymph node yield with the use of ICG fluorescence imaging. However, data on long-term outcomes including overall survival, disease-free survival, quality of life, and definitive tumor clearance were limited or not reported. While improved lymph node harvest suggests potential oncologic adequacy, no studies systematically assessed margin status or recurrence rates. Similarly, quality of life was not evaluated using standardized tools. Thus, the impact of ICG imaging and LCA skeletonization on long-term oncologic and functional outcomes remains unclear and warrants further investigation through well-designed prospective trials.

## 4. Discussion

Preserving optimal vascular supply during LAR is critical for ensuring safe anastomosis and minimizing postoperative complications, particularly AL.^[16]^ Anastomotic integrity relies heavily on adequate perfusion of the bowel ends; compromised blood supply is a major risk factor for leakage, which can lead to severe morbidity, prolonged hospitalization, and even mortality. In this context, intraoperative strategies that enhance perfusion assessment and preserve vascular anatomy have gained significant attention.^[17,18]^

This systematic review highlights that skeletonization of the LCA, particularly when performed in conjunction with real-time vascular assessment using indocyanine green (ICG) fluorescence imaging, offers tangible benefits in maintaining bowel perfusion. Skeletonization involves meticulous dissection of the perivascular tissue while preserving the artery itself, as opposed to high ligation of the IMA, which may jeopardize perfusion to the proximal limb of the anastomosis.^[19]^ This approach theoretically ensures dual vascular supply to the proximal colon via both the marginal artery of Drummond and the preserved LCA, thus supporting superior tissue oxygenation.^[20]^

ICG fluorescence angiography, which provides dynamic visualization of bowel perfusion in real time, offers objective parameters – such as time to fluorescence, fluorescence intensity, and slope of enhancement – that help assess vascular adequacy at the transection site.^[20]^ Across the reviewed studies, ICG angiography consistently demonstrated enhanced tissue perfusion when the LCA was skeletonized. These findings not only reduce reliance on subjective assessments (e.g., bowel color or arterial pulsation) but also support more precise and confident intraoperative decision-making regarding transection levels. Consequently, this may lead to fewer intraoperative changes to resection margins and improved outcomes.^[15]^

Additionally, some studies reported lower anastomotic leak rates in skeletonization groups compared to conventional high ligation, suggesting that improved perfusion translates into better anastomotic healing.^[8,9,11,12]^ Although these findings were not uniformly consistent across all studies – possibly due to variability in study design, patient populations, and ICG protocols – they nonetheless point to a potential protective role of LCA preservation in colorectal surgery.

From an oncologic standpoint, skeletonization of the LCA has raised concerns about the adequacy of lymph node harvest, particularly in cases of advanced or node-positive disease.^[21]^ However, emerging evidence suggests that when performed correctly, LCA skeletonization can achieve an oncologically acceptable lymphadenectomy in appropriately selected patients. Some authors advocate that adequate nodal clearance can still be attained through perivascular dissection along the LCA and IMA while maintaining blood supply to the anastomosis.^[22]^ Nonetheless, in patients with bulky lymphadenopathy or high-risk features (e.g., extramural vascular invasion or poor differentiation), high ligation may still be warranted to ensure radical oncologic resection.^[23]^

Another consideration is the technical demand of the skeletonization technique. It requires careful dissection and familiarity with vascular anatomy to avoid iatrogenic injury to the LCA or its branches.^[24]^ Moreover, standardized protocols for ICG usage – including timing, dosage, and interpretation criteria – are not universally established, which may limit reproducibility across institutions. LCA skeletonization, when guided by ICG fluorescence imaging, presents a promising approach that balances vascular preservation with oncologic safety.^[25,26]^ It appears particularly advantageous in patients undergoing LAR with mid- to low-rectal tumors where perfusion is critical and the risk of leakage is high. However, further prospective, randomized studies are needed to validate its impact on long-term outcomes, refine patient selection criteria, and establish standardized operative protocols.^[27,28]^

Despite these promising findings, several limitations must be acknowledged. First, the majority of the studies included were observational, with inherent biases such as patient selection, surgeon preference, and institutional protocols. Definitions of “skeletonization” varied, and the extent of dissection was inconsistently described, complicating direct comparisons across studies. In addition, ICG perfusion assessments were not standardized in terms of dosage, imaging timing, or quantification methods. Most outcomes focused on short-term perfusion and leakage rates, with limited data on long-term functional or oncologic results. Furthermore, the learning curve for both skeletonization and ICG use may influence outcomes, and these were not uniformly accounted for.

Future research should aim to establish standardized protocols for LCA skeletonization and ICG fluorescence imaging, including consistent definitions, perfusion metrics, and surgical endpoints.^[25]^ High-quality randomized controlled trials are needed to better determine the efficacy of this approach in reducing complications and improving outcomes. Studies should also investigate long-term effects on bowel function, cancer recurrence, and quality of life.^[29]^ Additionally, cost-effectiveness analyses may clarify the practicality of incorporating ICG routinely in colorectal surgery. As minimally invasive techniques and imaging technologies continue to evolve, the role of precision vascular preservation strategies such as LCA skeletonization is likely to become increasingly significant in optimizing colorectal surgical care.^[30]^

### 4.1. Complications, contraindications, disadvantages, limitations, and technical difficulties of ICG fluorescence imaging in colorectal surgery

While indocyanine green (ICG) fluorescence imaging offers clear advantages in assessing bowel perfusion and guiding surgical decisions, several limitations and potential issues must be considered. **Complications** related to ICG use are rare but include allergic reactions, particularly in patients with iodine sensitivity, although true anaphylaxis is exceedingly uncommon. **Contraindications** include known hypersensitivity to ICG or iodinated compounds, hepatic dysfunction (as ICG is excreted in bile), and severe renal impairment where dye clearance may be impaired. **Disadvantages** of the technique include added cost, the need for specialized imaging equipment, and a learning curve associated with interpretation of fluorescence signals. In terms of **limitations**, most current data are derived from observational studies with short follow-up, and the absence of standardized dosing, timing of injection, and fluorescence quantification methods reduces reproducibility and comparability across centers. Moreover, **technical difficulties** may arise from poor visualization due to obesity, mesenteric fat, or insufficient penetration of near-infrared light, which can limit the clarity of perfusion assessment. Additionally, the subjective interpretation of fluorescence intensity without quantitative perfusion metrics may lead to variability in surgical decision-making. Overall, while promising, ICG imaging should be integrated carefully, with awareness of its constraints and a need for further standardization and training.

## 5. Conclusion

Intraoperative indocyanine green (ICG) fluorescence imaging has demonstrated significant value in enhancing surgical precision and improving outcomes in colorectal cancer surgery. By providing real-time assessment of bowel perfusion, ICG use has been associated with reduced anastomotic leak rates, better lymph node yield, and improved intraoperative decision-making. The consistent findings across multiple studies support its integration into routine surgical practice, although further high-quality randomized trials are needed to validate its long-term oncologic benefits and cost-effectiveness.

## Author contributions

**Conceptualization:** Muhammad Zeeshan, Almaz Rehman, Muhammad Adeel.

**Data curation:** Almaz Rehman, Muhammad Adeel.

**Formal analysis:** Almaz Rehman, Muhammad Adeel.

**Investigation:** Sarim Rashid.

**Methodology:** Sarim Rashid, Muhammad Zeeshan.

**Project administration:** Sarim Rashid, Muhammad Adeel.

**Resources:** Sarim Rashid.

**Software:** Abida Parveen.

**Supervision:** Abida Parveen.

**Validation:** Muhammad Zeeshan, Abida Parveen.

**Visualization:** Abida Parveen.

**Writing** – **original draft:** Sarim Rashid, Muhammad Zeeshan, Muhammad Adeel, Abida Parveen.

**Writing** – **review & editing:** Sarim Rashid, Muhammad Zeeshan, Almaz Rehman, Muhammad Adeel, Abida Parveen.
